# A qualitative enquiry on the impact of mental illness stigma on caregiving role and experiences in Singapore

**DOI:** 10.3389/fpsyt.2024.1417514

**Published:** 2024-07-08

**Authors:** Wei Jie Ong, Chong Min Janrius Goh, Gregory Tee Hng Tan, Shazana Shahwan, Mythily Subramaniam

**Affiliations:** Research Division, Institute of Mental Health, Singapore, Singapore

**Keywords:** caregiver, Singapore, qualitative, mental illness, stigma, caregiving

## Abstract

**Introduction:**

Given that mental illness stigma is a common occurrence amongst people with mental illness and caregivers (CGs) can be a potential victim of stigma themselves, there is a need to examine caregivers’ perspective on the phenomenon. This study is part of a larger study which aims to qualitatively examine the concept of mental illness stigma amongst different stakeholders in Singapore.

**Methods:**

Focus group discussions (FGDs) were conducted amongst 21 informal caregivers to explore the experience of stigma encountered by them and their care recipients, and how it may implicate their caregiving experience. Both inductive and deductive thematic analyses were employed to analyze the data.

**Results:**

Three overarching themes of stigma encounters that may have implication on caregiving experience were identified: 1) Stigma within the family; 2) Structural stigma; and 3) Stigma by association. Experience of stigma within family (e.g., social exclusion and lack of understanding) limits the caregiving supports available to CGs. CGs also took up a mediating role between their care recipients and other family who may hold stigmatizing views. Witnessing their care recipients being subjected to structural disadvantages (e.g., employment, school, and mandatory conscription) can induce emotional stresses amongst CGs and motivate them to protest and seek redress on their behalf. Furthermore, encounters of stigma themselves (e.g., being judged or blamed for their loved one’s condition) also led to feelings of guilt and self-blame amongst the CGs.

**Discussion:**

These findings can aid the formulation of interventions in informing CGs on what to expect on their caregiving journey and supporting them in dealing with issues relating to stigma and highlight the importance of anti-stigma efforts in organizational settings such as schools, corporations, and government agencies.

## Introduction

Mental illness stigma is consistently and frequently experienced by people with mental illness (PMI) across different countries and cultures (1–9). Encounters of stigma can include being subjected to negative stereotypes (e.g., beliefs that PMI are dangerous or incompetent), attitudes (e.g., fear and aversion), and discriminatory behaviours (e.g., ostracization) (1–10). Furthermore, it can manifest in a multitude of contexts including social life, workplace, academic institutions and within the family (2, 7–9). Studies have documented that such experiences can result in a range of negative impacts amongst PMI (e.g., worsened psychiatric symptoms, lowered self-esteem, and reduced help-seeking, treatment adherence and quality of life) (11–15). Although often overlooked, people can also encounter stigmatizing experiences by being associated with PMI (e.g., family members, friends, and healthcare service providers) (16, 17).

Singapore is a multi-racial country state with a population of 5.92 million people, made up of three major ethnic groups, Chinese, Malay and Indian (18) As established by the nationwide Singapore Mental Health Study (SMHS) carried out in 2016, mental illness is not uncommon in Singapore, wherein 1 in 7 people would have experienced a mood, anxiety, or alcohol use disorder in their lifetime (19). In 2012, Singapore launched their Community Mental Health (CMH) masterplan, a paradigm shift that pivoted their focus from institutional psychiatric care to a greater emphasis on community-based care for PMI (20, 21). Given that Singapore is a collectivistic society which prioritises family ties, family members are often the customary caregivers (CG) of PMI in Singapore (22). Hence, the shift towards community-based care not only adds on to the caregiving load of family members but requires them to spend even more time with their care recipient.

Although providing care for a loved one can be an admirable and fulfilling task, it can also lead to strains on caregivers’ finances, and physical and mental health due to the challenges with meeting their care recipient’s needs (23–25). Studies have reported greater levels of burden amongst CGs of PMI as compared to CGs of people with chronic physical conditions (26, 27). Furthermore, it is consistently highlighted that CG burden can differ in impact and presentation depending on cultural influence. Chakrabarti (28) described that filial piety, a cultural notion that is common among the Asian population, could lead to increased CG burden and distress. Obligatory adherence and sacrifices in favor of the family’s values and attitudes towards supporting the family was highlighted as a specific dimension of filial piety that could negatively impact CGs (28). These findings highlight the susceptibility of CG burden to influences of social constructs.

Previous local studies have examined the different aspects of stigma such as occurrence, impact and the role of mitigating interventions and policies (8, 29–32). However, studies on caregivers’ perspectives on mental illness stigma and its impact on them is lacking despite them being arguably the next most knowledgeable people about their care recipients as well as being potential victims of stigma due to their association with their care recipients. Hence, given that mental illness stigma is also a social construct that manifests distinctly depending on culture, it is imperative to explore the possible ways in which mental illness stigma can implicate the experience of caregiving amongst local CGs (33).

Therefore, this study aims to explore encounters of mental illness stigma amongst CGs and its impact on their caregiving experience in Singapore. In this paper, the term “caregiver” will refer to informal caregivers and is defined as the person responsible for meeting the needs of their loved ones with mental illness. These are unpaid CGs who are not fulfilling their caregiving responsibilities as part of their professional duties (e.g., healthcare professionals).

## Materials and methods

### Study design and ethical approval

This research is part of a larger study that adopted an explorative qualitative approach to understand the concept of mental illness stigma among various stakeholders (i.e., the general public, person with mental illness (PMI), caregivers (CG) of PMI, healthcare professionals, and policymakers/influencers) in Singapore (8, 29–32). The study received ethical approval by the National Healthcare Group Domain Specific Review Board. Written informed consent was obtained from all respondents prior to their participation in the focus group discussions (FGD).

### Setting

The focus group discussions were conducted in meeting rooms at a neutral external site which ensured the confidentiality of participants and avoid biasing or priming responses related to the institution (which the authors belong to) during the FGD. Only study team members and respondents were allowed in the room.

### Respondents and sampling procedures

CGs were recruited, between July 2018 to November 2018, using convenience sampling. Majority of the respondents were recruited by contacting participants from previous studies who had agreed to be re-contacted for future research. Additional recruitment strategies included advertisement posters at the outpatient clinics of the Institute of Mental Health. All contacts prior to the FGDs were via phone and/or emails with the purpose of scheduling the session. No prior relationship between the study team members and the participants was established. Except a brief introduction (e.g., name and occupation) by the interviewer and note taker, no other information about the study team were shared with the participants. Participants were also given information about the purpose of the study limited to what was already written in the informed consent form. English is one of the official languages spoken, and is the common language used across the different ethnicities in Singapore. The literacy rates of residents aged 15 and over who speak either English only or English and one other language were 73.4%, 85.3% and 72% amongst the Chinese, Malay and Indian respectively (34). Hence, for this study, only respondents conversant in English were recruited.

#### Selection criteria

The study included Singapore citizens and permanent residents who were aged 21 years and above. Although it was advertised that the study sought caregivers of PMI with a mood or psychotic disorder, no confirmation on the diagnosis of their care recipients was made with their treating clinician or via medical records of the care recipient. The CGs had to provide social, physical, and/or financial support on a regular, personal basis, and were a family member or friend of the PMI. CGs who received financial incentives to provide care, such as foreign domestic workers, were excluded from this study.

#### Data collection

Data for this research study were collected through FGDs, where each session lasted approximately 1 to 1.5 hours. The total number of FGDs was determined based on data saturation, in other words, FGDs were conducted until no new themes or no new information on themes emerged from FGDs (35). This was determined via interim analysis and discussion amongst the study team members. A total of 5 FGDs were conducted, each comprising 4 to 9 respondents. All FGDs were conducted in English by experienced qualitative researchers with post-graduate qualifications (i.e., MS or SS) accompanied by a note-taker (i.e., CMJG, WJO or GTHT). Refer to **Table 1** for more information on the study team members. Background sociodemographic information (e.g., age, gender, ethnicity etc.) was collected individually prior to the commencement of the FGD, which was initiated with an ice-breaker introduction starting with the interviewer (i.e., name and role in the study) and amongst the respondents to situate them into the group. The FGDs were directed using a topic guide, which was developed by the study team (CMJG, SS, WJO, GTHT, and MS). The topic guide featured open-ended questions that investigated various experiences and encounters of mental illness stigma (enclosed as **Appendix A**). During the discussion, probing questions were also employed to further explore meanings and clarifications shared by the respondents. The questions were formulated based on Krueger et al’s (36) recommendations, which are - to be 1) relevant to the study’s objective; 2) neutral and simple to understand; 3) answerable by all participants; 4) open-ended; and 5) non-sensitive or intrusive. All study team members were involved in brainstorming of the questions. After the questions were consolidated, rephrased, and organized into a logically flowing order, the draft was circulated amongst the study team members for suggestions. The final decision for omitting any question was made by the lead investigator (MS). All 5 FGDs were audiotaped and transcribed verbatim for analysis.

**Table 1:** Interviewers’ and notes takers’ information.

Name	Gender	Credentials	Occupation	Experience
MS	Female	Doctorate of Philosophy	Research Director	Have attended several courses on qualitative research and are well versed with NVivo software for text analysis. Experienced with conducting qualitative studies and published multiple qualitative research papers.
SS	Female	Master’s degree	Research Manager	
WJO	Male	Bachelor’s degree	Research Assistant	
CMJG	Male	Master’s degree	Research Assistant	
GTHT	Male	Bachelor’s degree	Research Assistant	

#### Data analysis

The qualitative data collected was analyzed using thematic analysis with both inductive and deductive approaches (37). This approach involves the researchers’ familiarization with the data, coding (i.e., categorizing patterns), and theme development and revision. Data was analyzed using NVivo 12. The transcripts were distributed amongst the study team (CMJG, SS, WJO, GTHT, and MS) to independently identify preliminary nodes, which were further reviewed and refined to develop the finalized nodes and codebook used for coding. Inter-rater reliability was established prior to the commencement of coding the data, to ensure consistency within the study team. The researchers coded the same transcript independently before coming together to discuss and refine the codebook to foster like-mindedness until a satisfactory inter-rater reliability score was achieved (Cohen’s Kappa Score >0.75). A decision was made that two members of the team (CMJG and GTHT) would code the remaining FGD, where an inter-rater score of Cohen’s Kappa 0.81 was established. Nevertheless, throughout the process, the entire study team would meet to discuss the analysis when doubts in coding emerged. Codes were further refined and developed into themes that are reported in this paper. Verbatims reported in this paper were minimally edited to understand the vernacular in Singapore.

## Results

### Characteristics of respondents

A total of 31 caregivers (CG) were interviewed, and their age ranged from 22 to 73 years. Among the respondents, 21 of them were female and 10 were male. Majority of the respondents were of Chinese ethnicity (n = 21), identified as Christians (n = 10), married (n = 17), caring for a child with mental illness (n = 19), and whose care-recipient had a psychosis-related illness (n = 17). The duration of caregiving ranged from less than a year to 58 years (**Table 2**).

**Table 2:** Characteristics of respondents.

Characteristics		Frequency
***Sex***	Male	10
	Female	21
***Age (years)***	22 – 32	3
	33 – 43	3
	44 – 54	8
	55 – 65	11
	66 and above	6
***Ethnicity***	Chinese	21
	Malay	4
	Indian	5
	Eurasian	1
***Religion***	Christianity	10
	Taoism	1
	Buddhism	7
	Hinduism	2
	Islam	4
	Others	7
***Education***	Completed primary	2
	Secondary	2
	O/N Level/completed secondary	5
	A Level/completed pre-U or junior college	3
	Polytechnic diploma	5
	Other diploma	4
	University degree	4
	Post-graduate degree	4
	Others	2
***Marital status***	Single/never married	7
	Married	17
	Divorced/separated	4
	Widowed	3
***CG relationship***	Caring for a parent	4
	Caring for a child	19
	Caring for a sibling	4
	Caring for a spouse/partner	3
	Caring for a grandchild	1
***PMI diagnosis***	Psychosis-related	17
	Mood-related	6
	Anxiety-related	4
	Personality-related	1
	Unsure	2
	Co-morbid conditions	1
***Duration of caring (years)***	1 or less	6
	More than 1 to 10	17
	More than 10	7
	Unsure	1

### Themes

3 themes of stigmatizing experiences that have implications on the CGs themselves and their caregiving experience were identified (**Table 3**.).

**Table 3:** Themes on stigmatizing experiences that affect caregiving experience.

Themes	Frequency of Codes
Stigma within the family	39
Structural stigma	61
Stigma by association	20

### Stigma within the family

CGs had shared personal experiences where their care recipients were stigmatized by their immediate and extended relatives or witnessed similar experiences with other PMI. One frequent example was how the members of the family were not being understanding of the PMI and their condition.

“My son used to be very close to my daughter when they were young. After the onset of the illness, the symptoms he displayed, and it always becomes a big hoohaa. The whole family gets affected. Once this happened, my daughter kind of … she always put the blame on my son, says “why kor kor (older brother in Chinese dialect) behave this way? Then daddy mummy have to look after him.””– CG2

Another common example was when they purposefully avoided and excluded the PMI from their life.

“Like my youngest daughter got married and didn’t invite the sister. My son got married, also didn’t invite her, birthday party, or whatever party. She will be excluded because they worry that she will behave weirdly in a function and create a lot of trouble.” – CG31

A few of CGs had mentioned attempts to mediate the relationship between their care recipients and their less accepting family members in such situations.

“They get on, because I tried to reconcile. But she still feels that brother still behave this way, and then she doesn’t like to talk about it so she keeps a distance from the brother.” – CG2

“I say “can go (to sister’s wedding) never mind, they didn’t exclude you”, She said “they don’t like me I won’t go.” – CG31

CGs had also expressed that some extended and immediate family members were apprehensive towards their care recipients due to worries that they may “trigger” or be unable to manage the care recipients if problems arose. Furthermore, there was also the belief that there is little hope of recovery for PMI which resulted in the impression that providing care for PMI is a long term yet fruitless commitment. This had resulted in hesitation and contemplation towards providing additional caregiving supports to CGs.

“It’s because they worry that when my son acted up, they don’t know how to manage him. So they are kind of contemplating whether they can help you, take care of my son that kind of thing. So we were thinking whether is it stigma or because of their lack of understanding about the illness itself.” – CG02

“They don’t want to help, because they don’t want to take the responsibility. You know mental illness, in their case ah, mental illness they say cannot be cured like other sicknesses, this sickness will come off and on, and on and off, on, they … don’t want to take responsibility, they like to go on holidays. Even though one of my sister is working at a part time job, daytime she never does anything. Because of that I ask her for help, also don’t want to do.” – CG13

Several CGs had highlighted that they were reluctant to share their care recipient’s condition with other people including family members and relatives. This was due to various beliefs stemming from stigma such as apathy among the extended family, disclosure may lead to more stigmatizing encounters and judgement, and having someone with a mental health condition in the family being seen as shameful. Conflictingly, some CGs felt that concealing their care recipients is also considered as a form of stigmatization towards their loved ones.

“When it comes to friend, yes I am very secretive, I don’t tell everybody, I don’t trust you know? Erm because I’m scared of stigma, scared of how they will look at us.” – CG10

“I cannot share with our family. My family they all just don’t care.” – CG23

### Structural stigma

CGs also indicated that their loved ones often faced structural stigma in various situations. The most recurring mentions were on the need to declare one’s history of mental illness during applications for jobs, migration, scholarship, insurance and even going for a dental appointment. Many CGs believed that once their loved ones declared or when stakeholders (e.g., potential employers) learned of their condition, they would be disadvantaged.

“In school, in army or in certain big organisation, there is always a need to declare “are you mentally ill, do you have a mental problem?”, why is that in the form?” – CG4

“The moment you declare, out you go.” – CG31

CGs had also shared examples of lack of accommodation by schools, workplaces, and during mandatory conscription where their care recipients and PMI in general had experienced stigmatising attitudes and discrimination. In terms of schools, a few CGs had shared that some teachers were less understanding towards PMI - saw them as being lazy if they were not performing as well as the other students. A couple of CGs had also shared experiences where schools withdrew awarded scholarship or school placement after their care recipients were diagnosed with mental illness. A CG also complained that his daughter was rigidly penalized, and subsequently withdrew from the school due to their attendance protocol even though she had a psychiatrist’s memo explaining that her absence was due to her condition.

“One of the teacher said, ‘the teacher’s job is to teach’. I think he meant teach a normal student. So, if he cannot perform, they just chuck him aside. Or just give him just average (an average grade), just pass.” – CG22

“My daughter was admitted to XXX junior college. One of the top junior college. Second year she started to have bipolar already, what did the school say? Take her out. Finish.” – CG31

There were multiple mentions on how PMI are at a disadvantage when seeking employment and may face difficulties in maintaining their job. This was attributed to the negative perception that PMI are incompetent and have high absentee rate due to medical reasons as well as how most corporates are business-driven.

“We may see it as they cannot perform, always take medical leave. We think that they are not able to do their work well, then we start to judge.” – CG9 (with working experience in the corporate world)

“It’s very competitive and ultimately it’s like what you (CG20) said, you’re looking for an employee who is going to benefit the company.” – CG21

A CG also shared how her daughter’s supervisor used derogatory languages to comment about her poor performance.

“It’s irrelevant. After the customer is gone, they (supervisors) ask “may I know how old is your mother?… Your mother must have given birth to you when she was very old, that’s why you get this Down syndrome.” – CG14

CGs also revealed about the lack of accommodation towards PMIs during the mandatory conscription where Singaporean males, 18 and above are to serve two years in government service, more specifically in the military, police, and civil defence forces. Some examples include superiors being unaccepting of PMIs, treating them poorly, unwilling to trust them with tasks or trying to discharge them from the service. A CG had also mentioned that his son was exempted from mandatory conscription despite the latter’s interest in serving.

“They just get him to sit down there to flip newspaper, not even dare to get him … simple jobs like filing yes, but like, since you want him to be there, just let him finish two (years)… like do nothing you know, like they don’t trust him to complete the task.” – CG9

“Then he was exempted from mandatory conscription, but I didn’t tell my son, I know he will blame me, he will regret, because his dream is to be a police officer.” – CG10

“A lot of officers there feel that ‘why did you let this person in?’, they want to discharge him.” – CG9

One CG had also shared her experience where her daughter was treated harshly by the police due to her suicide attempt. Instead of being assessed by a psychiatrist, she was handcuffed, shackled, and locked up at the police station.

It was very cruel for a mum to have to see her daughter handcuffed … she was also shackled in her feet, and it was very bad … And I said that “look she has mental illness, she has this depression illness, she has a long history of suicidality.” So I am just wondering why is she not being assessed by the IMH doctor, and instead, being waited you know locked up in the cell and then has to wait for the medical officer.” – CG11

Many CGs had attempted to protest or seek help against such experiences (e.g., contacting the relevant ministries, and approaching news agencies to publish about the stigmatizing experience). However, these efforts usually went in vain.

“While she was not around, the rest of the officers don’t know how to take care of him. So they were yelling, screaming and when he went to the medical institution for check-up. No, cannot (keep him), discharge him. So we went all the way to the higher order. Ok never mind, we talk to the senior officials whatever, you accepted him, then why now say cannot?” – CG2

“I wrote letter to the authorities in education. They dare not reply me. I wrote this letter to forum, XX paper … they dare not publish. My experience with this, with this discrimination, I was very angry. I was even willing to pay money to advertise my experience, in XX paper, they refused to accept.” – CG31

Such stigma encounters by their care recipients often led to considerable emotional stress to CGs. A CG also expressed his concern regarding his care recipient’s future due to the structural stigma PMI faced.

“So throughout this 10-15 hours ordeal, I was actually … at that time I became crazy myself.” – CG11

“When she went to poly, that was when I … and it really affected me so much. Because I can see the understanding is not there.” – CG29

“But I tell my sister how I am going to tell him that his dream uh won’t come true you know, so I am very sad about this thing, then the school, some like … you know what happen to his future I am thinking. If everybody know that his sickness, his condition, then how, how will he lead his life. What he’s going to be. These are my worries.” – CG10

Institute of Mental Health (IMH), the only tertiary care psychiatric institute in Singapore was often associated with negative connotations. As described by the CGs, people harboured many false ideas about the hospital (e.g., it is like a prison and “mad house”), and it can be a shocking news to some if one was admitted in it.

“Mental illness is very much misunderstood. And that people have got utterly false ideas of what goes on in IMH. And so, if you were to say your loved one was admitted into IMH, then it will be something that will be shocking for other people.” – CG17

A CG had shared the challenges she faced with bringing her care recipient to IMH follow-ups and activities due to his negative perceptions about the hospital.

“After seeing the doctor he was reluctant to take the medicine, so he Googled, he read … I don’t know what kind of article he read. So I brought him to IMH and the first experience was no good for him. He heard screaming, shouting, and then he says “mummy I am not crazy, why you bring me here? And then we went into the doctor’s room to see the doctor. He don’t want to talk to the doctor, he don’t want to see the doctor, he went out.” – CG10

### Stigma by association

CGs themselves could also become victims of stigma just by being associated with their care recipients. A couple of CGs alluded that people have the general impression that family members of PMI are also mentally unwell to some degree. Another CG also mentioned that children who have family members with mental illness were more likely to be bullied.

“You tell them that your family has this issue, the next time they look at you like you also have that issue, when you do something wrong.” – CG27

“So if a child has relatives that have mental illness, then the child will be bullied, everybody will start bullying that child.” – CG26

In addition, several CGs had consistently shared that having a mental illness is often seen in Asian culture as a form of retribution or punishment from gods for the family’s wrongdoings. Hence, having someone with a mental illness in the family is perceived as shameful and can be damaging to the family’s social standing. Such cultural beliefs also led to CGs, especially parents, being judged or blamed for their loved one’s condition. As a result, several CGs and their family members had experienced guilt and self-blame for their care recipients’ conditions.

“It’s because in Chinese we believe that probably you have done something bad, it may be in the past you know, that’s why bad karma, you have done something bad, that’s why you know, it’s a retribution to the … retribution to the family that you have somebody who is sick. So that’s one reason why people see that as shameful.” – CG2

“I don’t know if that’s just an Asian thing, like “oh what happened to your child, what did you do, how did you…, maybe you didn’t take care of yourself when you were pregnant” or “you weren’t strict enough with her”. It was just really horrible”. – CG21

## Discussion

This study identified 3 themes of stigma experiences that may affect CG’s caregiving experience: 1) stigma within the family; 2) structural stigma; and 3) stigma by association.

CGs in our studies shared experiences of stigma within the family such as other family members not being understanding towards the care recipients as well as excluding them from their life. There are an abundance of studies that consistently documented experiences of abandonment and exclusion of PMI by their family members (38). Liegghio (39)’s qualitative paper has also found that many younger siblings with either an older brother or sister with a mental illness showed difficulties in making sense of their older sibling’s condition and thus perceived them negatively. In response to such experiences, a couple of CGs in our study had attempted to mediate the relationship between their care recipients and the less accepting family members. As per many Asian countries, Singapore is a collectivistic society where interpersonal ties are prioritized (40). Furthermore, it was also suggested that in the Chinese culture, people often perceive family and social relationships as a representation of their greater self (41, 42). Hence, given that Singapore is a multiracial country made up of a blend of Asian origin races, and with Chinese being the majority ethnic group, it is not surprising that the CGs in our study tried to preserve harmony within the family. However, as highlighted by Jeon and Madjar (43), CGs are often caught struggling to strike a balance between meeting their care recipients’ needs and attending to their other family members. Another qualitative study also found that siblings of PMI often felt neglected by their parents due to the lesser attention paid to them (44). As suggested, constant adjustment and adaptation are key components to maintain family functioning when living with a PMI (45). Hence, CGs should be tactful when conflicts occur to ensure harmony in the family. However, such considerations are often complex, adding to the taxing role of a CG.

CGs in our study had also mentioned that their extended and immediate family may feel apprehensive toward their care recipients due to their worries with not knowing how to interact with someone with a mental illness. In conjunction with the belief that PMI have poor prognosis, many of them hesitated or were reluctant to provide caregiving support. Furthermore, due to anticipated stigma, CGs in our study had also expressed reluctance in sharing about their care recipients’ conditions with others including immediate and extended family members. These findings demonstrated the impact of stigma on limiting the availability of caregiving support to main CGs and avenues to confide their concerns. Studies have postulated that it is common for CGs to experience loneliness and isolation when providing care for their care recipient, which can have a detrimental effect on their physical and mental health, and health behaviours (e.g., increase tobacco use) (46–50). Hence, this finding further stresses on the importance of combating mental illness stigma and increasing mental health literacy amongst the general public.

Family psychoeducation which often involves educating family members of PMI of their symptoms, prognosis, recommend treatments, ways to better interact with them and strategies on forming a conducive relationship have been found to be effective in improving family’s QOL and, their attitudes and beliefs towards the PMI and mental illnesses (51–54). This could be a possible solution for combating stigma within the family. Furthermore, such interventions should also include strategies on how to mediate family conflicts due to stigma and better equip CGs with skills to play a bridging role between family members as well as information on possible channels for CGs to seek support and emotion regulating exercises to help cope with emotional distress from caring for their loved ones.

CGs had also pointed out instances of structural stigma that their care recipients have personally experienced or were often subjected to. This included the need to declare one’s history of mental illness, which often put PMI at a disadvantage, as well as the lack of accommodation that they encountered in various organizational settings (i.e., school, places of employment, and mandatory conscription). CGs can experience negative emotions and stress when their care recipients were faced with such encounters. Disadvantages faced by PMI in school and employment are widely documented in literature across different cultures (8, 55–57). However, stigma encountered during mandatory conscription is more relevant to Singapore and countries where citizens (most of the time males) are required to serve a mandatory period in government services such as in the military, police, and civil defence forces. Studies done overseas have provided evidence of mental illness stigma in the military settings, such as being perceived as weak in a masculinity preferred setting, malingering, and as unfit or incompetent to perform duties, resulting in being undervalued by superiors (58, 59). Furthermore, performance during the conscription is often used in social and occupational context to prospectively gauge one’s resilience and competency in Singapore (60). Hence, failure to complete or being exempted from it may further place PMI at disadvantages in various aspects of life and deny them from leading autonomous lives. However, there is a need to acknowledge that the stressful nature of serving conscription (e.g., physically demanding training and performing of duties) may lead to worsening of psychological symptoms (61). Therefore, it is important to strike a balance between accommodating to PMI and managing the possible risks that may occur during their service period.

Multiple qualitative studies conducted across different countries, have also found that CGs experienced concerns and worries for the future of their care recipients in terms of self-sufficiency (62–64). CGs in our study also shared this concern as a result of the structural stigma. Given that most of the CGs in our studies were parents of their care recipients, the prevalence of structural stigma may had led to such concerns being more pronounced amongst them (8). Hence, this may explain the stress that CGs experienced in response to the structural disadvantages and hardship that their care recipients face in the different domains of life that may affect their future livelihood.

Some of the CGs also mentioned protesting or help-seeking on behalf of their care recipients in response to the structural stigma encountered. However, similar to what was found in literature, such efforts were often disregarded (56). Plausible reasons for the persistence of structural stigma include the difficulties in documenting such occurrences, especially if it is unintentional, and the possible discounting by people in position of power who may see accusations of injustice as threatening to their own privileges (56). This shows how individuals’ efforts alone are not enough to combat structural stigma. As such, greater efforts and understanding within organizations and government agencies are needed to identify processes that may disadvantage PMI. CGs in our studies also highlighted that there are many negative connotations and false ideas associated with the mental health institution which may deter care recipients from seeking treatment and rehabilitation.

Apart from signing the United Nations Convention on the Right of Person with Disabilities (UNCRPD; an international agreement that aims to ensure equal rights for people with disabilities) in 2013, the Singapore government has recognised the need for mental illness anti-stigma efforts in the recent years (65). Some of these efforts include the introduction of guidelines for fair and progressive employment practices by the Tripartite Alliance and the complementary Workplace Fairness Legislation that is soon to be passed in 2024 (66, 67). There was also the decriminalizing of attempted suicide in 2020 which aims to steer survivors towards recovery rather than burdening them with legal proceedings (62). With that said, continuous effort is needed to formulate new policies, monitor, and revise existing ones, and ensure collaborative efforts from different stakeholders to ensure their effectiveness. Furthermore, although Singapore has been steering towards community-based care in terms of mental healthcare, mental health institutions and general hospitals still play an important role in providing PMI a holistic and integrated care, alongside community-based stakeholders (68). Therefore, apart from making information available on community based care such as treatments and avenues of supports, information on employment opportunities and redressal of unjust treatment must be made readily available to PMI and CGs. There is also the need to improve perceptions towards the mental health institute to instill confidence amongst people toward the services they provide.

Consistent with earlier literature, CGs in our study also reported being subjected to stigma by association. There exists a belief that family members of PMI too suffer from mental illness, and children of PMI are often likely to be bullied. Furthermore, CGs were sometimes judged and blamed for their care recipients' conditions due to the common Asian belief that having a mental illness is a form of punishment from God, a form of retribution or karma due to the wrongdoings of members of the family. CGs in our study had shared experiences of guilt and self-blame for possibly contributing to their loved ones’ conditions through past actions which was similarly found amongst CGs in Moses (69)’s study (e.g., bad parenting, oversight of care recipients’ mental health, and negative family environment). This finding demonstrates the influence of cultural and societal beliefs, specifically causal beliefs of mental illness, in shaping the manifestation of stigma. Although many studies had focused on the adverse impact of non-biomedical explanatory models of mental illness (e.g., delayed help seeking and greater stigma, as per our finding), it has been postulated that having multiple explanatory models can potentially help PMI and CGs cope with the distress from the long and unpredictable course of the illness and undesirable treatment outcomes (70, 71). Hence, instead of perceiving different explanatory models as competing, it may be useful to incorporate the various perspectives in designing culturally appropriate anti-stigma campaigns and interventions (72).

There are some limitations to this study. Participants in our study may be subjected to social desirability bias given the presence of other participants in a FGD setting. It is possible that they may have withheld certain opinions. To minimize the bias, participants were assured that information shared during the FDGs will be kept strictly confidential. Furthermore, participants who agree to take part may have strong views about mental illness stigma or had been significant impacted by them. Therefore, they may be inherently different from those who refused to took part in the study. As this qualitative study is one of the first to explore the impact of stigma on caregivers, future studies may seek to develop quantitative scales incorporating the theme expressed in the current study to better capture the experience of stigma amongst CGs and its specific impact on their caregiving journey.

To conclude, the findings of the study have demonstrated that mental illness stigma can affect the role and experience of the CGs, regardless of whether the stigma experience was encountered by their care recipients or themselves. Encounters of stigma within the family can affect the availability of additional caregiving support and outlets for CG to confide in. It also impelled CGs into taking up mediating roles to improve the relations between their care recipients and less accepting family members. Moreover, the disadvantages and unjust encounters faced by PMI due to structural stigma can also lead to emotional distress amongst the CGs and motivate them to protest and seek redress on behalf of their loved ones. Finally, CGs are often seen as being responsible for their care recipients’ conditions due to cultural beliefs, which can result in feelings of guilt and self-blame. This study’s findings can aid the formulation of interventions in informing CGs on what to expect on their caregiving journey and supporting them in dealing with issues relating to stigma. The disadvantages faced amongst PMI due to stigma would not only affect their livelihood and future, but also increases the caregivers’ burden in the long run.

## Data availability statement

The datasets presented in this article are not readily available because respondents did not consent to share their data publicly. Requests to access the datasets should be directed to Prof Mythily Subramaniam, mythily@imh.com.sg.

## Ethics statement

The studies involving humans were approved by Domain Specific Review Board - National Health Group. The studies were conducted in accordance with the local legislation and institutional requirements. The participants provided their written informed consent to participate in this study.

## Author contributions

WJO: Writing – original draft, Writing – review & editing. CMJG: Writing – review & editing. GTHT: Writing – review & editing. SS: Writing – review & editing. MS: Writing – review & editing.
