# Supplementary material for: A qualitative enquiry on the impact of mental illness stigma on caregiving role and experiences in Singapore
Source: Front Psychiatry. 2024 Jul 8;15:1417514. doi: 10.3389/fpsyt.2024.1417514 (PMC11262131; doi:10.3389/fpsyt.2024.1417514)
Supplement: Supplementary file 1 [file DataSheet_1.docx]

**Appendix A – Focus group discussion topic guide caregiver version**

**FGD Guide for Qualitative Study to Explain the Concept of Stigma**

**Caregiver**

**Instructions for Team**

1. **Only facilitator and main minute-taker to stay in the room.**
2. **Switch on audio-recorder.**
3. **Provide participant with sheet of paper and pen for writing.**

**FGD Guide for Caregiver**

**Introduction to FGD**

Welcome to today’s focus group discussion and thank you for your interest and time in participating in this discussion today. I am ________, the facilitator of today’s focus group discussion and I am with the Institute of Mental Health (IMH) conducting research in mental health.

Also with me here today are my other colleagues (introduce them)

First of all, thank you for agreeing to take part in this research study. Your participation is very important for meeting our study objectives and your inputs will greatly help us to understand stigma towards mental illness.

**General Ground Rules**

**To be written up on flip chart/board:**

**1. Confidentiality**

**-use names on tags**

**-keep personal details and discussion within this room**

**2. Be Respectful**

**-no side conversations**

**-take turns to speak**

**3. We want to hear from you!**

**-no right or wrong answers**

**4. Turn handphones to silent**

We have already spoken to you about the purpose and requirements of the study. As you are aware, we will be audio-recording this conversation. This is just to help us get all the details of today’s conversation. Once we have recorded all the necessary details of the discussion we will be destroying the audio recording. So please be assured that confidentiality will be maintained at all times and your name or other identification will not be revealed in any documents. I hope that everyone here feels comfortable with the taping.

I would like to request you to speak clearly and use only the names that each of you have written on your name tag. I would request all of you to not discuss personal details or talk about today’s specific discussion with people outside the group. Let us all maintain confidentiality and respect each other’s views during the session. I would also like to request you not to have side conversations when someone else is speaking, interrupt each other or talk over each other as we may not be able to record clearly what is being said. Let’s make this a lively yet respectful discussion.

This study aims to understand what mental illness stigma is, how and why it might arise in Singapore, why people stigmatize against people with mental illness and how this stigma can be counteracted and reduced. I hope we can have an open discussion today.

We want you to hear from you. We want to know *your* thoughts, opinions, experiences and observations of this topic. Please be assured that this is a safe space to speak your mind. There are really no right or wrong answers, so please feel free to share them with us. I am sure you know much more than me, and I would like to learn more from you.

Before we begin I would like to once again assure you that you have the right to withdraw from the study or refuse to provide any information that you feel uncomfortable with during the course of our discussion.

Lastly, I would like to request you to kindly turn your hand phones to silent mode to minimize distraction during the discussion. I would also like to point out that the toilets can be found (give directions).

Today’s discussion will last for one and a half hours and we will end by **XX** pm. Please feel free to help yourself to the refreshments provided.

Do you have any questions before we start?

## FGD guide for Caregiver

**I. Self-introductions (5mins)**

Let’s start by getting to know each other. Tell me your name (first name, pseudonym), a little bit about yourself and one other thing about you. You can choose from any of these categories:

**To write on flip chart**

**Self introductions**

- **Name**

**Choose:**

- **Your hobby**
- **Fact about you**
- **Favourite movie**
- **Favourite food**

**II. Thoughts about Mental Illness and Stigma (25mins)**

- Can you list down 5 thoughts that come to mind when you think about people with mental illness?
- Based on your own understanding, can you describe the mental illness that the person you are providing care for is suffering from?
- Can you tell us in your own words what “Stigma” means to you?

**III. Dependent and Mental Illness Stigma**

- Has the person whom you are currently providing care faced any form of stigmatizing^1^ behaviors?
- How would these stigmatizing behaviors affect the person you are providing care for?
  - (probe) Have you done anything to combat or overcome this?
- Have you ever tried to conceal the fact that the person you are providing care for has a mental illness?

**IV. Caregiver and Mental Illness Stigma**

- Have you faced any stigmatizing behaviors yourself for providing care for someone with a mental illness? If yes, can you elaborate on them?
- How would these stigmatizing behaviors affect you in general?

**V. General Questions on Stigma**

- What do you think could be some of the causes for stigma towards people with mental illness to arise in Singapore?
- Some people believe that the culture plays a role in stigma, what are your thoughts? If so, how does it impact on stigma?
- To what extent do you think that mental illness could be due to some fault of the person?

**VI. Anti-stigma efforts**

- Have you heard of any anti-stigma campaigns?
- How do you think stigma towards people with mental illness can be reduced?

We have reached the end of the interview. Is there anything more you’d like to tell me that we have not already discussed?

Do you have any questions for me about anything we have discussed?

**VII. Summarise the FGD for participants.**

^1^to use words used by caregivers to describe stigma
